# Replacement of HA-MRSA by CA-MRSA Infections at an Academic Medical Center in the Midwestern United States, 2004-5 to 2008

**DOI:** 10.1371/journal.pone.0092760

**Published:** 2014-04-22

**Authors:** Michael Z. David, Adriana Cadilla, Susan Boyle-Vavra, Robert S. Daum

**Affiliations:** 1 Department of Medicine, University of Chicago, Chicago, Illinois, United States of America; 2 Department of Pediatrics, University of Chicago, Chicago, Illinois, United States of America; Rockefeller University, United States of America

## Abstract

We noted anecdotally that infections designated as health care-associated (HA-) MRSA by epidemiologic criteria seemed to be decreasing in incidence at the University of Chicago Medical Center (UCMC) after 2004. We compared MRSA patients seen at any site of clinical care at UCMC and the isolates that caused their infections in 2004-5 (n = 545) with those in 2008 (n = 135). The percent of patients with MRSA infections cultured > 2 days after hospital admission decreased from 19.5% in 2004-5 to 7.4% in 2008 (p = 0.001). The percent in 2004-5 compared with 2008 who had a hospitalization (49.1% to 26.7%, p = 0.001) or surgery (43.0% to 14.1%, p<0.001) in the previous year decreased. In 2008 a greater percent of patients was seen in the emergency department (23.1% vs. 39.3%) and a smaller percent both in intensive care units (15.6% vs. 6.7%) and in other inpatient units (40.7% vs. 32.6%) (p<0.001). The percent of patients with CA-MRSA infections by the CDC epidemiologic criteria increased from 36.5% in 2004-5 to 62.2% in 2008 (p<0.001). The percent of MRSA isolates sharing genetic characteristics of USA100 decreased from 27.9% (152/545) to 12.6% (17/135), while the percent with CA-MRSA (USA300) characteristics increased from 53.2% (290/545) to 66.7% (90/135). The percent of infections that were invasive did not change significantly. Our data suggest that HA-MRSA infections, both by epidemiologic and microbiologic criteria, relative to CA-MRSA, decreased between 2004-5 and 2008 at UCMC.

## Introduction

The epidemiology of methicillin-resistant *Staphylococcus aureus* (MRSA) changed dramatically in the first decade of the twenty-first century with the emergence of genotypically novel community-associated (CA-) MRSA strains [1]. CA-MRSA strains were first noted in the late 1990s as causes of skin and soft tissue infections (SSTIs) as well as severe invasive syndromes in people with no known previous exposure to a health care setting. Subsequently, a single genetic background, identified as USA300 by pulsed-field gel electrophoresis (PFGE), became the most common cause of SSTIs in U.S. emergency departments (EDs) in 2004 [2] and 2008 [3]. USA300 was the most common genetic background of MRSA causing bacteremia in 23 U.S. hospitals in 2009–2010 [4]. In surveillance at 12 laboratories in Minnesota, CA-MRSA infections defined by an epidemiologic criterion accounted for 33% of all MRSA infections in 2005, an increase from 11% in 2000 [5].

USA300 has progressively increased as a proportion of MRSA isolates causing infections cared for at large medical centers. As a consequence, the overall number of MRSA infections has increased [6]. This has resulted from 4 epidemiologic trends. First, the incidence of outpatient and ED MRSA SSTIs [7], [8] and the incidence of CA-MRSA infections resulting in hospitalization [9] has increased in the past decade. Second, USA300 has increasingly been the cause of nosocomial infections and infections among people with healthcare exposure [10]. Third, there is evidence that there were fewer invasive MRSA infections in the U.S. among patients with recent health care exposure [11]. Fourth, during the past decade, among all MRSA infections in several U.S. populations, there has been an increase in the percent that are classified epidemiologically as CA-MRSA [5], [12]–[14].

We noted anecdotally that infections designated as health care-associated (HA-) MRSA by epidemiologic criteria seemed to be decreasing in incidence at the University of Chicago Medical Center (UCMC) after 2004. Previously, we assessed the clinical and molecular epidemiology of MRSA infections at UCMC from July 1, 2004 to June 30, 2005 [15]. At that time, we found that CA-MRSA infections, whether defined by epidemiologic or microbiologic criteria, were very common among inpatients and outpatients in the clinic and the ED among both adults and children. Here, we compare the results of that study with the clinical and microbiologic characteristics of MRSA patients and their MRSA isolates at UCMC in 2008. Our hypothesis was that between 2004-5 and 2008, there was a major shift in the overall molecular epidemiology of MRSA infections at our center, with a greater percent of infections caused by MRSA strains bearing SCC*mec* type IV, a greater percent of isolates carrying the genes for the Panton-Valentine leukocidin (PVL), and a higher percent of strains having the ST8 multilocus sequence type (MLST), characteristics, when taken together, that identify USA300 [16].

## Methods

### Ethics Statement

The study was approved by the University of Chicago Biological Sciences Division Institutional Review Board (IRB). The IRB approved the following consent process. In-person consent was not feasible because the study was performed without specific funding, and patients, in the majority of cases, were no longer inpatients at our institution at the time of enrollment. Telephone consent was obtained utilizing an IRB-approved text read to potential subjects; for children <7 years of age, consent was obtained from a parent or guardian. For children 8 to 18 years of age, in addition to consent from a parent or guardian, telephone assent was obtained from the patient. Patients who refused consent were not enrolled. Consent was documented on a paper consent form for each enrolled subject except in cases when the subject (or a parent or guardian for a child) could not be reached despite at least 5 attempts. In cases when telephone consent could not be obtained, a waiver of consent was granted by the IRB, and note was made of this in the study records.

UCMC is comprised of pediatric and adult hospitals serving the population on the south side of Chicago as well as tertiary care patients from elsewhere in the midwestern United States, with 26,288 admissions, 82,828 ED visits, and 388,277 outpatient visits in fiscal year 2008. UCMC follows the guidelines of the Health Care Infection Control Practices Advisory Committee of the Centers for Disease Control and Prevention [17]. Also, in March 2008 UCMC began a nasal screening for MRSA carriage, using a PCR-based test, of all patients admitted to an intensive care unit, as mandated by Illinois state law. Carriers identified in this way were placed on contact precautions.

### Bacterial Isolates

We began prospectively collecting and storing MRSA isolates obtained by the UCMC Clinical Microbiology Laboratory on November 1, 2003. MRSA was isolated from 839 unique patients cultured as part of an evaluation for an infection in January 1 - December 31, 2008. A random 20% sample of these patients and their isolates was selected (n = 168). The first MRSA isolate obtained from each selected subject was chosen for analysis except when the first isolate represented asymptomatic colonization, in which case the first isolate from the patient's clinical infection was used.

### Medical Record Review

Medical records were reviewed for each enrolled subject to assess age, gender, race, type of insurance, location of care when the MRSA culture was obtained, clinical MRSA syndrome, the presence or absence of an indwelling vascular catheter, comorbid medical conditions, and any previous MRSA infection or colonization. As in our previous study [15], each MRSA clinical syndrome was assigned to a syndrome category (SSTI, osteomyelitis or septic arthritis, bacteremia and endocarditis, urinary tract infection [UTI], pneumonia or other). All infections were separately classified as invasive if the isolate was obtained from a normally sterile site, and all other infections were considered non-invasive. Invasive infections included pneumonia, bacteremia, endocarditis, sepsis, osteomyelitis, septic arthritis, pyomyositis, peritonitis, necrotizing fasciitis and other deep-seated skin and soft tissue infections, and cholecystitis. Subjects with isolates representing asymptomatic colonization were excluded from further analysis. Data collected for the 2008 subjects were identical to those collected for the 2004-5 patient cohort previously described at UCMC [15] except that, in 2008, data on previous surgery were collected for the year prior to the MRSA infection. For the 2004-5 data, history of surgery was assessed only for 6 months prior.

### Microbiological Methods

For each MRSA isolate, antimicrobial susceptibilities to erythromycin, ciprofloxacin, rifampin, gentamicin, trimethoprim-sulfamethoxazole, and vancomyin were determined by Vitek (Vitek 2, bioMérieux Vitek, Inc., Durham, NC). However, the D-zone test was performed on all isolates found to be clindamycin susceptible and erythromycin resistant on single-agent testing, according to NCCLS Guidelines [18].

### Genotyping

For each studied isolate, multilocus sequence typing (MLST) was performed as described [19]. Detection of the Panton Valentine leukocidin (PVL) genes was performed by PCR as described [20]. The SCC*mec* type in the isolates was determined by a panel of PCR assays that allowed for the determination of the *mec* and ccr complex [21], [22].

### Criteria for CA- and HA-MRSA

Eight criteria commonly used to distinguish CA- from HA-MRSA infections were applied to each patient and his or her MRSA isolate. Clinical criteria included the (1) the 48-hour criterion, (2) CDC epidemiologic definitions for CA-MRSA and HA-MRSA [11], and (3) SSTI as the clinical syndrome. (1) By the 48-hour criterion, an infection was considered to be CA-MRSA if it was cultured as an outpatient or <2 days after admission to the hospital, and other infections were considered to be HA-MRSA. (2) A MRSA infection was considered to be HA-MRSA by the CDC epidemiologic definitions if, in the year prior to culture, the subject had surgery, hospitalization, hemodialysis or a stay in a long-term care facility, if an indwelling vascular catheter was in place at the time of culture, or if the subject was an inpatient hospitalized for >2 days at the time of culture. Otherwise, the subject was considered to have a CA-MRSA infection [11]. The CDC has a third category, the health-care-associated community-onset (HACO) group, and we include this in the group of HA-MRSA infections by the CDC criteria. Patients and their MRSA isolates were also separately classified as CA-MRSA by isolate characteristics, including (4) the presence of the PVL genes, (5) the presence of SCC*mec* type IV, (6) the ST8 MLST genotype, (7) clindamycin susceptibility, and (8) non-multidrug resistance [non-MDR, defined as resistance to ≥2 tested non-β-lactam antibiotic drugs]), as done in our previous study [15].

### Statistical Analysis

For each clinical and microbial characteristic, subjects and their isolates in 2004-5 [15] were compared with those in 2008. Data were compared by the chi-square, Fisher Exact, or t-test, as appropriate (Stata 11, College Station, TX). The percent pairwise concordance for every possible pair of the 8 criteria for CA-MRSA was tabulated.

## Results

Among 168 patient-isolates designated for study, 149 were enrolled. Upon chart review, 135 (90.6%) were determined to have had an infection and 14 (9.4%) only had MRSA colonization. Further analyses were limited to the 135 enrolled subjects with clinical infections. Comparing patients with MRSA infections in 2004-5 and 2008, there was no significant difference in the distribution of clinical syndromes, pediatric vs. adult age groups, or race, but the subjects differed significantly by distribution of types of insurance coverage, with the percent of uninsured and privately insured higher in the 2008 patient cohort and the percent publicly insured lower (p = 0.001) (Table 1). Although this change was statistically significant, the magnitude of the change was not great, and it is difficult to determine the importance of the finding.

**Table 1:** Demographic and clinical characteristics of patients with methicillin-resistant *Staphylococcus aureus* infections at UCMC in 2004-5 and 2008.

	2004-5, N = 545, (%)	2008, N = 135, (%)	p-value^a^
**Clinical syndrome**			0.1
Bacteremia, endocarditis, or sepsis	63 (11.6)	15 (11.1)	
Osteomyelitis or septic arthritis	33 (6.1)	4 (3.0)	
Pneumonia	46 (8.4)	8 (5.9)	
Skin and soft tissue infection	354 (65.0)	103 (76.3)	
Urinary tract infection	22 (4.0)	1 (0.7)	
Other^b^	27 (5.0)	4 (3.0)	
**Age Group**			0.3
Pediatric (<18.0 years)	200 (36.7)	56 (41.5)	
Adult	345 (63.3)	79 (58.5)	
**Gender**			0.9
Male	268 (49.2)	67 (49.6)	
Female	277 (50.8)	68 (50.4)	
**Race**			0.5
African American	406 (74.5)	96 (71.1)	
White	84 (15.4)	19 (14.1)	
Latino	11 (2.0)	4 (3.0)	
Native American	3 (0.6)	0	
Unknown or other	41 (7.3)	16 (11.9)	
**Type of insurance**			0.001
Public assistance	378 (69.4)	76 (56.3)	
Private	132 (24.2)	42 (31.1)	
Uninsured	19 (3.5)	15 (11.1)	
Unknown	16 (2.9)	2 (1.5)	
**Presence of risk factors for HA- MRSA** c			
Inpatient culture obtained >48 hrs after admission	106 (19.5)	10 (7.4)	0.001
Hospital stay, past year	225 (41.3)	36 (26.7)	0.002
Surgery, past 6 months	209 (38.4)	19 (14.1)	<0.001
Hemodialysis, past year	35 (6.4)	5 (3.7)	0.3
Indwelling catheter	69 (12.7)	8 (5.9)	0.03
Stay in long-term care facility, past year	16/290 (5.5)	8 (5.9)	0.9
**Location of care**			<0.001
Intensive care units	85 (15.6)	9 (6.7)	
Other inpatient units	220 (40.7)	44 (32.6)	
Emergency department	126 (23.1)	53 (39.3)	
Outpatient	112 (20.6)	29 (21.5)	
**CDC Criteria for Infection Type**			<0.001
CA	199 (36.5)	84 (62.2)	
HA	346 (63.5)	51 (37.8)	
**Previous MRSA isolation**			
UCMC laboratory report	58 (10.6)	21 (15.6)	0.1

a The p-value was determined using the χ-square or the Fisher exact test, as appropriate; for mutually exclusive categorical variables a single test was performed to compare the distribution of the data for 2004-5 with the data for 2008.

b Includes cholecystitis, conjunctivitis, peritonitis and upper respiratory infection.

c Denominators for HA-MRSA risk factors in 2004-5 exclude those interviewed patients who answered that that they did not know information requested of them and those patients about whom risk factor information could not be determined from chart review. Information regarding a stay in a long-term care facility for 2004-5 patients was determined only for those patients lacking another health-care risk factor for HA-MRSA infection. For 2008 patients, this was evaluated for all 135 enrolled.

### Clinical characteristics of patients

The percent of MRSA patients with clinical characteristics of CA-MRSA infections increased in 2008 compared with the 2004-5 cohort. The percent of patients with MRSA infections who were cultured > 2 days after hospital admission (i.e., the 48-hour criterion) decreased from 19.5% in 2004-5 to 7.4% in 2008 (p = 0.001). The percent in 2008 compared with 2004-5 having recorded exposures to the health care setting decreased significantly. These included hospitalization (41.3% to 26.7%, p = 0.002) or surgery (38.4% to 14.1%, p<0.001) in the previous year. In 2004-5 compared with 2008, there was a lower prevalence of patients who had received hemodialysis in the past year and of patients with an indwelling catheter at the time of culture, although these differences were not significant. There was no significant change in the percent of patients with a history of a stay in a long-term care facility in the year prior to culture (p = 0.9). The site of care at UCMC differed (p<0.001), with a greater percent of patients in 2008, compared with 2004-5, receiving care in the ED (23.1% versus 39.3%) and a smaller percent in intensive care units (15.6% versus 6.7%) and in other inpatient units (40.7% versus 32.6%) (Table 1), demonstrating a shift in the site of care for MRSA patients from inpatient to outpatient settings.

**Figure 1:**
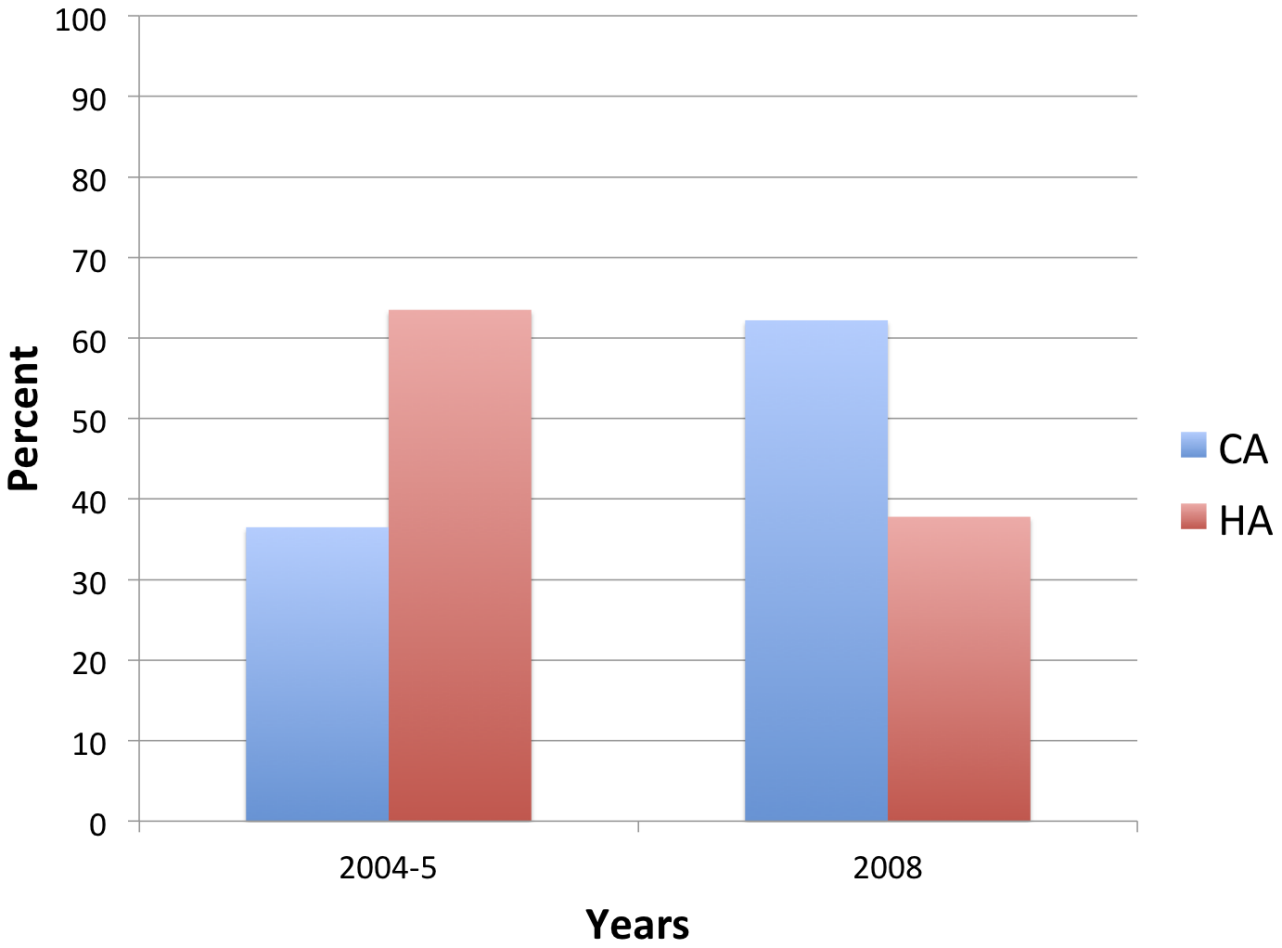
Percent of MRSA patients in 2004-5 and 2008 classified as CA-MRSA and HA-MRSA infections by the CDC epidemiologic definition. The percent that were HA-MRSA decreased from 63.5% (346/545) to 37.8% (51/135) (p<0.001), demonstrating a shift in the balance in the site of onset of MRSA infections from the healthcare setting to the community.

The percent of MRSA patients who would be classified as having CA-MRSA infections by the CDC definition increased from 36.5% in 2004-5 to 62.2% in 2008 (p<0.001). Thus, there was a significant shift, even by this stringent criterion for CA-MRSA infections, in the focus of MRSA epidemiology from people with previous exposure to the health care setting to those without such exposures (Figure 1). The percent of patients who had an SSTI diagnosis increased from 65.0% (354/545) in 2004-5 to 76.3% (103/135) in 2008 (p = 0.01).

### Genetic characteristics of isolates

In 2004-5 there were 12 MLSTs (10 defined STs and 2 single-locus variants of defined STs) represented among the MRSA isolates studied, and in 2008, there were 14 types, demonstrating little evidence of changing diversity in the repertoire of genetic MRSA backgrounds. However, the frequencies of different MLSTs varied in 2004-5 and 2008 (p<0.001).

By microbiological criteria, the percent of isolates that would be categorized as CA-MRSA increased in 2008 compared with 2004-5. The presence of the PVL genes (58.2% versus 76.3%, p<0.001) and SCC*mec* type IV carriage (65.7% versus 79.4%, p = 0.006), both markers of CA-MRSA strains in the U.S., increased in 2008 compared with 2004-5. The percent of ST8 isolates (a marker for USA300 CA-MRSA isolates) increased from 58.9% to 71.1%, and the percent that were ST5 (a marker for USA100 HA-MRSA isolates) decreased from 31.2% to 15.6%. The percent of isolates sharing the genetic characteristics of USA100 (ST5, SCC*mec* type II, PVL-negative) decreased from 27.9% (152/545) to 12.6% (17/135). The percent of isolates with the CA-MRSA USA300 strain type's characteristics (ST8, SCC*mec* type IV, PVL-positive) increased from 53.2% (290/545) to 66.7% (90/135). Interestingly, comparing isolates from 2004-5 and 2008, there was not a significant change in susceptibility to most tested antimicrobial drug among those tested. The exception was clindamycin, for which the susceptibility increased significantly in 2008 when compared with 2004-5 (Table 2). The percent that were MDR decreased from 34.7% (189/545) in 2004-5 to 21.5% (29/135) in 2008 (p = 0.003).

**Table 2:** Genotypic and phenotypic characteristics of methicillin-resistant *Staphylococcus aureus* isolates at UCMC from 2004-5 and 2008.

	2004-5 N = 545, (%)	2008 N = 135 (%)	p-value
**Clonal Complex type/MLST type** a			<0.001
*Clonal complex 1*			
1	21 (3.9)	1 (0.7)	
*Clonal complex 5*			
5	170 (31.2)	21 (15.6)	
5slv^b^	1 (0.2)	0	
105	3 (0.6)	2 (1.5)	
231	14 (2.6)	2 (1.5)	
1730	0	1 (0.7)	
2113	0	1 (0.7)	
2114	0	1 (0.7)	
*Clonal complex 8*			
8	321 (58.9)	96 (71.1)	
8slv^b^	2 (0.4)	0	
72	1 (0.2)	0	
683	0	4 (3.0)	
2511	0	1 (0.7)	
2515	0	2 (1.5)	
2516	0	1 (0.7)	
2522	0	1 (0.7)	
*Clonal complex 22*			
22	6 (1.1)	0	
2512	0	1 (0.7)	
*Clonal complex 30*			
30	0	0	
36	3 (0.6)	0	
*Clonal complex 59*			
59	2 (0.4)	0	
*New ST* c	1 (0.2)	0	
**PVL gene carriage**			<0.001
Positive	317 (58.2)	103 (76.3)	
Negative	228 (41.8)	32 (23.7)	
**SCC** ***mec*** ** type**			0.003
II	180 (33.0)	27 (19.9)	
IV	358 (65.7)	108 (79.4)	
Other^d^	7 (1.3)	0	
**Antibiotic resistance to** e			
Ciprofloxacin	230 (42.2)	48 (35.6)	0.2
Clindamycin (total)	220 (40.4)	36 (26.7)	**0.003**
Clindamycin, Vitek testing	176 (32.3)	34 (25.2)	0.1
Clindamycin, D-Test +	44 (12.4)	6 (4.4)	0.6
Erythromycin	500 (92)	122 (90.4)	0.6
Gentamicin	30 (5.5)	2 (1.5)	0.05
Trimethoprim/sulfamethoxazole	2/320	3 (2.2)	0.2
Rifampin	9 (1.7)	1 (0.7)	0.7
Vancomycin	0	0	N/A

a MLST clonal complex designations vary with time. The clonal cluster designations indicated here were accurate at the conclusion of 2008.

b *slv*, single locus variant with no assigned multilocus sequence type (MLST).

c 1-31-1-1-12-1-40 denote the allotypes of 7 genes (*arcC, aroE, glpF, gmk, pta, tpi,* and *yqiL*) that determine this undefined MLST type.

d 7 MRSA isolates from 2004-5 had multiple or no *ccr* genes and were thus considered nontypable.

e Includes intermediate susceptibility results as resistant. In some cases, isolates were not tested for clindamycin, ciprofloxacin, erythromycin susceptibility, and the number tested are indicated in the table as the denominator for each relevant category.

In 2004-5 there were 317/545 (58.2%) PVL-positive isolates, which were ST8 (n = 290), ST1 (n = 19), ST5 (n = 5), ST8 single-locus variants (n = 2), and ST59 (n = 1). In 2008, there were 103/135 (76.3%) PVL-positive isolates, which were ST8 (n = 92), ST683 (n = 4), ST2515 (n = 2), and a single isolate each of ST1, ST5, ST2511, ST2516, and ST2522. In 2004-5 SCC*mec* type IV was carried by 358/545 (69.7%) isolates, which were ST8 (n = 316), ST1 (n = 20), ST5 (n = 11), ST22 (n = 6), ST59 (n = 2), ST8 single-locus variants (n = 2), and ST72 (n = 1). In 2008, SCC*mec* type IV was carried by 108/135 (79.4%) isolates, which were ST8 (n = 93), ST683 (n = 4), ST5 (n = 3), ST2515 (n = 2), and a single isolate each of ST1, ST2511, ST2512, ST2514, ST2516, ST2522.

Even when limiting the analysis to HA-MRSA infections by the CDC epidemiologic criteria, the percent that were caused by USA100-like (i.e., ST5, SCC*mec* type II-bearing and PVL-negative) and USA300-like (i.e., ST8, SCC*mec* type IV-bearing and PVL-positive) changed in the study period. In 2004-5 there were 41.4% (138/333) USA100-like and 36.3% (121/333) USA300-like isolates, and in 2008, these values changed to 27.5% (14/51) and 43.1% (22/51), respectively, suggesting a nearly significantly decreased role of USA100 (p = 0.06) among epidemiologically defined HA-MRSA infections. Among CA-MRSA infections defined by the CDC criteria, 6.6% (14/212) in 2004-5 and 3.6% (3/84) in 2008 were caused by USA100-like isolates.

### Characteristics of infections: Invasive and non-invasive

The percent of infections that were invasive did not change significantly. In 2004-5, 27.0% (147/545) of MRSA infections were classified as invasive compared with 23.7% (32/135) in 2008 (p = 0.4). Invasive HA-MRSA infections (defined by the CDC criteria) increased, but not significantly, as a percent of all HA-MRSA infections in 2004-5 compared with 2008 from 39.9% (133/333) to 49.0% (25/51) (p = 0.2). The percent of invasive CA-MRSA infections among all CA-MRSA infections (by the CDC criteria) did not differ significantly in the two study periods, 6.6% (14/212) in 2004-5 and 5.7% (7/135) in 2008 (p = 0.6).

Among USA300-like strains obtained from patients who had CA-MRSA infections by the CDC epidemiologic definition in 2004-5, 4.1% (7/169) were invasive, and in 2008, 7.4% (5/68) were invasive (p = 0.5), a change that was not significant. Similarly there was not a significant change among USA300-like strains causing invasive HA-MRSA infections by the CDC definition, with 24.8% (30/121) in 2004-5 and 22.7% (5/22) in 2008 being invasive (p = 0.9).

### Concordance of criteria for CA-MRSA

Tables 3 and 4 show the percent concordance of any combination of 2 out of 8 studied epidemiologic and microbiologic criteria for CA-MRSA often used to distinguish CA- and HA-MRSA infections. The CDC definition for CA-MRSA had approximately 50% concordance with other criteria often used to identify CA-MRSA infections in 2004-5. The CDC definition in 2008 performed better in identifying patient-isolates that would be considered CA-MRSA infections. For example, in 2004-5 (Table 3), 67.3% of isolates had SCC*mec* type IV and were CA-MRSA by the CDC definition or lacked SCC*mec* type IV and were HA-MRSA by the CDC definition; in 2008 (Table 4), this percent increased to 71.9%. Similarly, the concordance between the CDC definition for CA-MRSA and the presence of PVL in an infecting isolate rose from 69.4% in 2004-5 to 78.5% in 2008. This increase in concordance may be due to the greater prevalence of CA-MRSA among all MRSA infections in 2008 and the corresponding decrease in the percent that were HA-MRSA infections.

**Table 3:** Percent concordance of 8 paired criteria for CA-MRSA, 2004-5.

Criterion	48-hour	Clindamycin susceptibility	SCC*mec* IV	Non-MDR	PVL+	MLST 8	SSTI
Clindamycin susceptibility	69.5						
SCC*mec* IV	73.8	89.5					
Non-MDR	74.5	93.2	93.8				
PVL+	69.5	88.3	92.5	90.2			
ST8 (MLST)	69.9	89.4	90.1	90.3	89.4		
SSTI	71.2	73.8	76.2	77.6	74.9	73.4	
CDC definition	58.3	67.5	67.3	67.7	69.4	66.8	65.9

**Table 4:** Percent concordance of 8 paired criteria for CA-MRSA, 2008.

Criterion	48-hour	Clindamycin susceptibility^a^	SCC*mec* IV	Non-MDR	PVL+	MLST 8	SSTI
Clindamycin susceptibility^a^	70.9						
SCC*mec* IV	83.0	85.5					
Non-MDR	80.0	95.5	91.1				
PVL+	79.2	89.0	94.1	93.3			
ST8 (MLST)	73.3	80.0	86.0	82.9	88.2		
SSTI	82.2	75.5	74.1	78.5	80.7	76.3	
CDC definition	69.6	73.6	71.9	74.8	78.5	72.6	75.6

a N = 110.

## Discussion

Our data suggest that in 2008, the proportion of MRSA infections that were HA-MRSA, both by epidemiologic and microbiologic criteria, decreased compared with 2004-5 at UCMC. Most strikingly, there was a significant decrease in the percent of MRSA infections cultured from inpatients more than 2 days after admission, in the percent that had surgery or a hospital stay in the previous year, and in isolates having the ST5 background, the most common background of U.S. HA-MRSA strains (USA100).

At the same time, the percent of all MRSA infections that were invasive did not change during the two periods studied. The shift in molecular epidemiology of MRSA infections, with more USA300-like and fewer USA100-like isolates causing infection in 2008 compared with 2004-5, suggests that both of these genetic backgrounds are associated with invasive disease. This pattern, i.e., a shift in genetic backgrounds without a significant shift in the percent of infections that were invasive, also suggests that the development of an invasive infection may be more likely driven by patient characteristics and exposures than by characteristics of the bacteria causing infections although such patient characteristics were not assessed in our chart reviews.

Patients with MRSA infections over time were increasingly seen in the ED and less often in the inpatient setting at our center. This shift in site of care for MRSA infections occurred with no significant change in the overall distribution of the types of clinical syndromes caused by MRSA comparing 2004 with 2008. While there was a non-significant increase in the percent of SSTIs among patients in the 2008 cohort compared with the 2004-5 cohort, the percent that had bacteremia, endocarditis, or sepsis or other invasive infections was similar. This suggests that while the focus of onset of MRSA infections moved to the community in 2008, the shift was not simply because of an increase in the percent of SSTIs among MRSA infections.

There were 2 dominant genetic backgrounds at UCMC in 2004-5 and 2008. Comparing these 2 time periods, there was a shift in their relative prevalence, with a lower percent of ST5 and a greater percent of ST8 isolates, i.e., from USA100 to USA300. The most common HA-MRSA genetic background, characteristically ST5 with SCC*mec* II and lacking the PVL genes, decreased from about one-third to less than one-sixth of all MRSA isolates that we sampled at our medical center. The USA300-like isolates, i.e., ST8 carrying the SCC*mec* type IV element and PVL+, caused both CA- and HA-MRSA infections by the CDC epidemiologic criteria, as noted in a few other U.S. centers [10], [23]. Few CA-MRSA infections were caused by USA-100-like isolates.

We found that as USA300 has become an increasingly prevalent genetic background among MRSA infections, USA100 became less so. In 2008, at our center less than one-quarter of HA-MRSA infections defined epidemiologically were caused by USA100-like isolates. This trend has been shown in few previous studies. While CDC Active Bacterial Core surveillance documented a decrease in invasive HA- and HACO-MRSA infections between 2005–2008, it did not report on non-invasive MRSA infections [11], as we have done.

A limitation to our study is our decision to use a 20% sample of all unique patients with a MRSA infection in 2008 at our medical center. It is possible that this number of patients is not an adequate representation of all patients in 2008. However, our patient sample was randomly selected, and with this sample, we demonstrated a significant change in the percent of infections caused by CA- compared with HA-MRSA infections at our medical center, no matter which of the many criteria are used to distinguish them.

As HA-MRSA strains become increasingly infrequent in the U.S., it is possible that we will enter an era in which USA300 is nearly the exclusive cause of MRSA infections in inpatient and outpatient settings. This is concerning because isolates of this background have been shown to be easily transmissible and virulent, potentially exacerbating infection control efforts. Further surveillance is warranted for genotypic change and for changes in antimicrobial susceptibility among MRSA isolates causing clinical infections at U.S. medical centers.
